# Bystander effects elicited by single-cell photo-oxidative blue-light stimulation in retinal pigment epithelium cell networks

**DOI:** 10.1038/cddiscovery.2016.71

**Published:** 2017-02-06

**Authors:** Masaaki Ishii, Bärbel Rohrer

**Affiliations:** 1 Department of Ophthalmology, Charleston, SC 29425, USA; 2 Department of Neurosciences, Division of Research, Medical University of South Carolina, Charleston, SC 29425, USA; 3 Ralph H. Johnson VA Medical Center, Division of Research, Charleston, SC 29401, USA

## Abstract

‘Bystander effect’ refers to the induction of biological effects in cells not directly targeted. The retinal pigment epithelium consists of hexagonal cells, forming a monolayer interconnected by gap junctions (GJs). Oxidative stress initiated in an individual cell by photostimulation (488 nm) triggered changes in reactive oxygen species (ROS), Ca^2+^ and mitochondrial membrane potential (*ψ*_m_). The Ca^2+^ signal was transmitted to neighboring cells slowly and non-uniformly; the ROS signal spread fast and radially. Increased Ca^2+^ levels were associated with a loss in *ψ*_m_. GJ blockers prevented the spreading of the Ca^2+^, but not the ROS-related signal. The GJ-mediated Ca^2+^ wave was associated with cell death by 24 h, requiring endoplasmic reticulum–mitochondria Ca^2+^ transfer. Ensuing cell death was correlated with baseline Ca^2+^ levels, and baseline Ca^2+^ levels were correlated with pigmentation. Hence, local oxidative stress in a donor cell can trigger changes in certain connected recipient cells, a signal that required GJ communication and an ROS-Ca^2+^ dual-hit. Finally, damage apparently occurred in susceptible cells, which correlated with baseline Ca^2+^ levels.

## Introduction

The bystander effect in biology refers to the phenomenon of induction of biological effects in cells that are not directly targeted, and has been studied in great detail in the radiation field.^1^ Available experimental data typically fall into two categories. The first category describes experiments that involve the transfer of medium from treated cells, which results in a biological effect in untreated cells. In media transfer experiments, the effects may be due to a molecule secreted by the treated cells, or even the transfer of exosomes.^2^ The second category describes experiments that involve the ability to treat specific cells, with biological effects studied in their neighbors. For these experiments, the means of communication is via gap junctions (GJs). In connected cells, small molecules that pass through GJ, such as glutathione, NADH, ATP/ADP, calcium, miRNA or even glucose, might be mediating the communication (e.g., Davidson *et al.*^3^ and Valiunas *et al.*^4^).

GJs chemically and electrically connect cells in tissues, including the heart, the central nervous system, as well as many epithelial cell sheets such as the lung and retinal pigment epithelium (RPE). Coupling can be relatively fast, acting in time frames of microseconds to seconds. Our interest lies in understanding intercellular communication in the context of stress or injury. GJ communication may help cells withstand low/moderate stress by metabolic cooperation, in which coupled cells can share metabolites such as NADH or ATP;^5^ however, under high levels of stress, GJ coupling might lead to a depletion of metabolites, or worse, the transmission of damaging compounds, which may lead to cell death.^6^

Herein we investigated the phenomenon of the bystander effect in RPE cell monolayers. The RPE is composed of a single layer of hexagonal, highly pigmented cells and forms part of the blood–retina barrier. It is a main target in disease processes, typically triggered by oxidative stress. Its barrier function is mediated by tight and adherence junctions, whereas intercellular communication is mediated by GJ containing connexin43 and connexin46.^7^ As the RPE is a highly coupled network, any individual cell will be significantly affected by the behavior of its neighbors. However, the susceptibility of a given cell to bystander signal from its neighbor is dependent upon its prior metabolic history and mitochondrial health. Here we examined cell–cell communication using live-cell imaging in response to photo-oxidative stress. In particular, we focused on three different but related questions. First, we examined the characteristics of stress-initiated induction and transfer of reactive oxygen species (ROS) and calcium ions (Ca^2+^) from the stimulated to connected neighboring cells, as well as their relationship to mitochondrial membrane potential (*ψ*_m_). Second, in longer-term imaging experiments, we examined the induction and spreading of apoptotic cell death throughout the RPE networks induced by photo-oxidative stress in a single cell, as well as the role of endoplasmic reticulum (ER)–mitochondria Ca^2+^ transfer in the control of apoptosis. And finally, third, we aimed to identify metabolic markers associated with susceptibility of a cell to a bystander signal.

## Results

### Photo-oxidative stress in ARPE-19 cells

**Figure 1:**
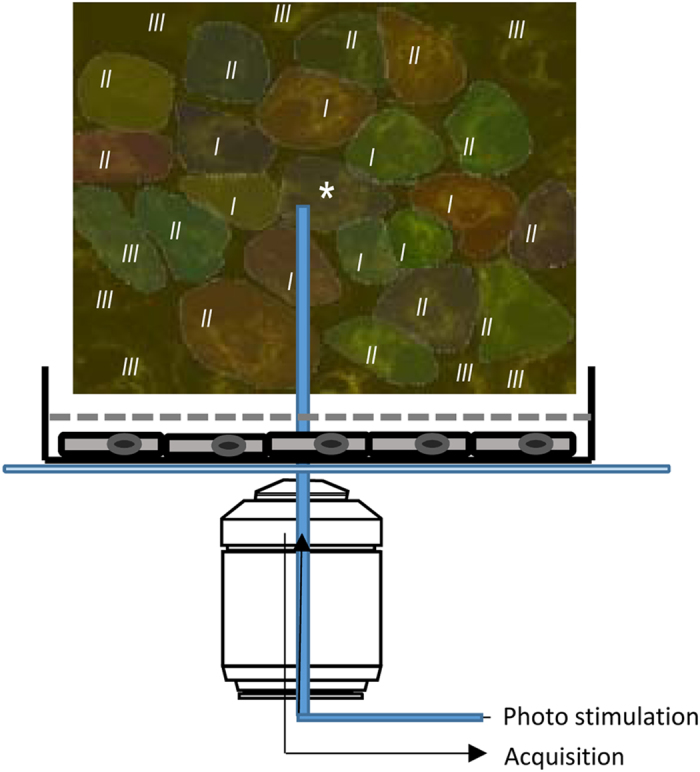
Experimental setup to trigger photo-oxidative stress. RPE were grown on glass-bottom dishes for >3 weeks after which they formed a monolayer. Tight and gap junction formation was confirmed, using antibodies against zonula occludens-1 (ZO-1), occludin and connexin 43 (Supplementary Figure S1). Image acquisition and photostimulation were performed using an upright microscope (Nikon Eclipse Ti, Tokyo, Japan) with a spinning disk confocal microscope (UltraView VoX 3D Live Imaging System). For all experiments, a central cell (labeled by an asterisk, *) was stimulated using a 488 nm laser spot (1 Hz; each flash lasting 20 ms). The six primary cells surrounding the central cells (I) and the corresponding potential 12 secondary cells (II) are indicated.

**Figure 2:**
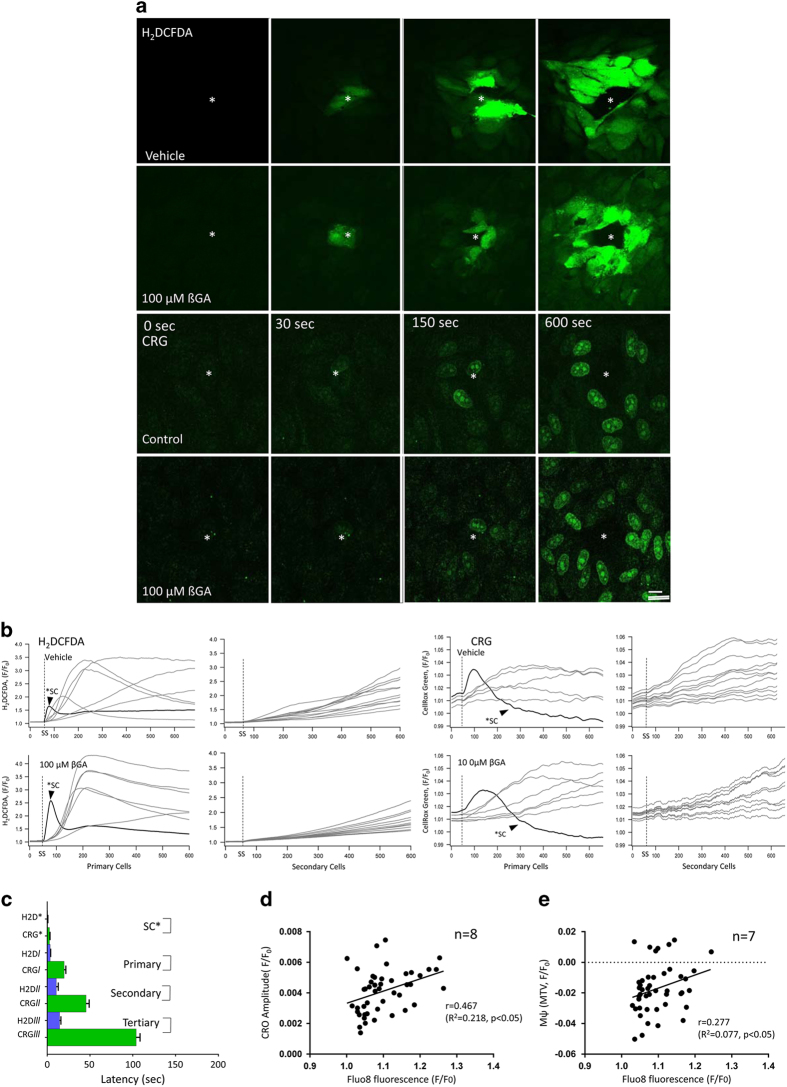
Photo-oxidative stress triggers an oxidative stress signal that is transmitted to all neighbors. Using the setup described in Figure 1, ROS was detected using CellRox Green (CRG) and 2′,7′-dichlorodihydrofluorescein diacetate (H_2_DCFDA) fluorescence using time-lapse imaging. (**a**) H_2_DCFDA (upper two rows) and CRG (bottom two rows) signals increased in the central, stimulated cell (asterisk, *) and the spread to the surrounding cells in a radial manner, independent of the presence of the gap junction blocker, 18-*β*-glycyrrhetinic acid (*β*GA). The CRG signal indicates oxidation by ROS (hydroxyl radicals and superoxide anions) in the cytoplasm and subsequent binding to DNA, whereas H_2_DCFDA detects hydrogen peroxide in the cytoplasm. (**b**) Fluorescence intensity profiles were plotted over time for the H_2_DCFDA (left-hand columns) and CRG (right-hand columns) both in the presence (bottom graphs) and absence (top graphs) of *β*GA for the photostimulated cell (SC) as well as the primary and secondary surround cells, respectively. The onset of the photostimulation is indicated (stippled line). The oxidative stress signal is transmitted to surround cells irrespective of the presence of *β*G, although the rise in signal is slower and the signal amplitude is smaller if gap junction communication is blocked. (**c**) Latencies for the rise in CRG and H_2_DCFDA (H2D) follow a consecutive pattern in all cells: stimulated, primary, secondary and tertiary surround cells. (**d**) To determine whether the elicited oxidative stress response (CRO) is correlated with the baseline calcium signal (Fluo8), the respective signals were plotted and significant cross-correlation determined (*r*=0.467 and *P*<0.05). (**e**) Similarly, significant cross-correlation was determined between the baseline calcium signal (Fluo8) and the elicited change in mitochondrial membrane potential (*ψ*m; TMRM; *r*=0.277 and *P*<0.05).

**Figure 3:**
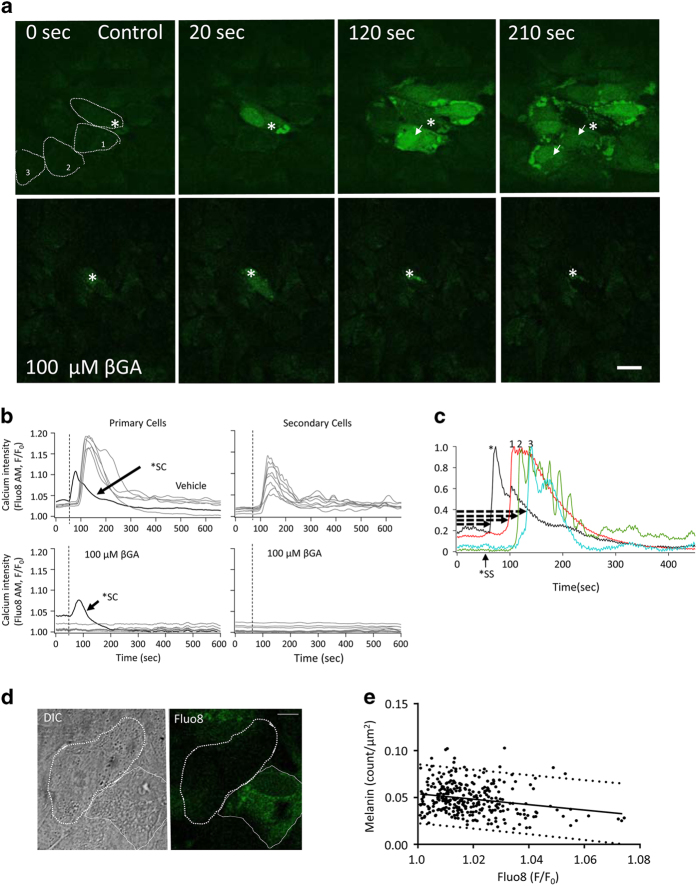
Photo-oxidative stress triggers a calcium signal that is transmitted to a subset of neighbors via gap junction communication. (**a**) Using the setup described in Figure 1, intracellular calcium (Fluo8 AM; green) changes were imaged in response to exposing a central cell to oxidative stress by photostimulation over the mitochondrial area (asterisk, *). Time-lapse images of the Fluo8 changes observed in control (upper) and 18-*β*-glycyrrhetinic acid (*β*GA)-treated monolayers (bottom) are shown. In control cells, Fluo8 changes were observed first in the central (20 s), followed by primary surround cells (120 s). The signal consists of a cytoplasmic followed by a mitochondrial signal (Figure 4). *β*GA treatment prevents the spread of the Fluo8 signal to neighboring cells. The same results were observed for 1-octanol (data not shown). Scale bar, 100 *μ*m. (**b**) The calcium signal (Fluo8) as imaged in the stimulated, primary and secondary surrounding cells of monolayers treated with vehicle (control) and *β*GA were plotted with reference to the onset of the photostimulation (stippled line), revealing both a transient and a long-term change in calcium. (**c**) The time delay of the calcium signal (indicated by arrows) is indicated to demonstrate the transfer of information through the network from central, primary, secondary to tertiary surrounding cells. (**d**) Differential interference contrast images (left) and Fluo8 signals (right-hand image) of two cells with higher (dashed line) and lower (solid line) melanin granule content are shown. Scale bar, 10 *μ*m. (**e**) A negative correlation between melanin granule density and Fluo8 intensity was identified. The solid line indicates the linear regression line and the dashed lines the 95% confidence interval.

**Figure 4:**
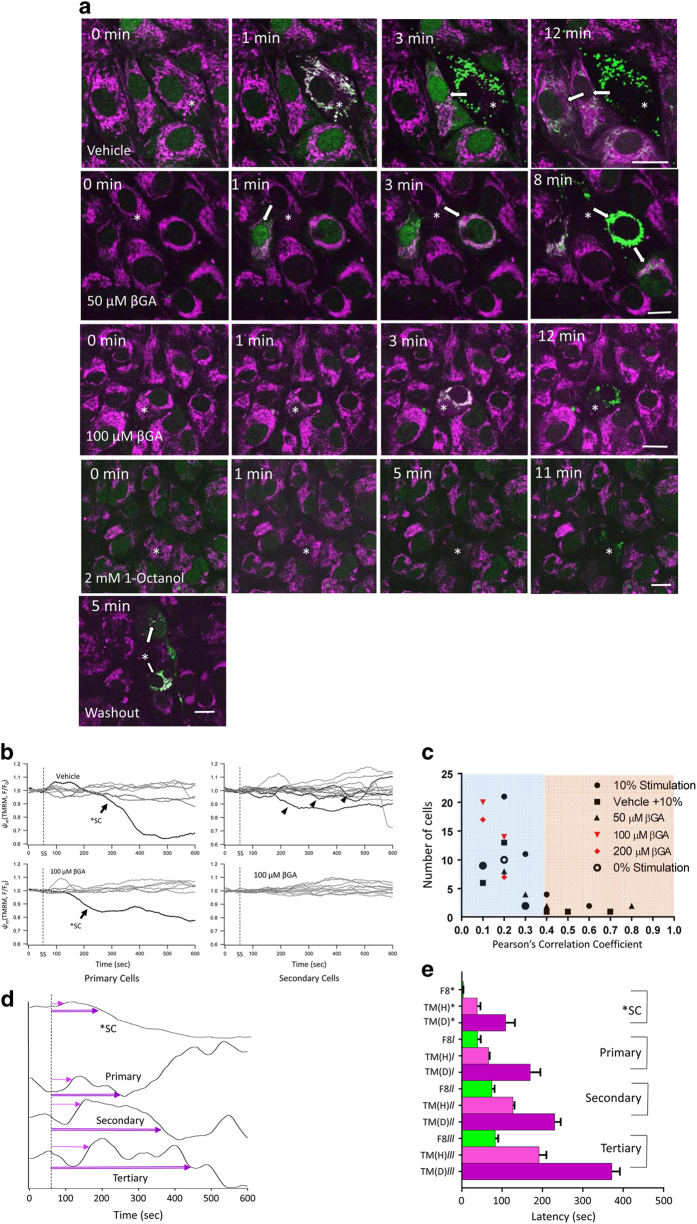
Calcium wave elicited in central cell by photo-oxidative stress triggers calcium uptake into the mitochondria. Figure 3 demonstrated that the calcium signal in the RP network consists of a rise in cytoplasmic calcium followed by an uptake into cellular compartments. (**a**) To confirm that calcium was taken up into the mitochondria, cells were imaged using Fluo8 AM (calcium; green) and tetramethylrhodamine methyl ester (TMRM; a dye readily taken up by active mitochondria; purple). In vehicle-treated cells, cytoplasmic calcium increases led to a Fluo8 fluorescence increase in mitochondria in the stimulated cell (SC; 1 min). The calcium signal was transferrable from the SC (asterisk, *) to adjacent neighboring cells (arrows), leading to an increase in cytoplasmic followed by mitochondrial uptake (co-localization indicated by white). This signal transfer could be inhibited by treatment with 100 *μ*M 18-*β*-glycyrrhetinic acid (*β*GA), but not 50 *μ*M, or 2 mM 1-octanol. Importantly, signal transfer could be recovered after a 5 min washout of *β*GA or 1-octanol. Scale bar, 20 *μ*m. (**b**) As the retention of TMRM within the mitochondria is dependent upon the mitochondrial membrane potential (*ψ*_m_), the TMRM signal was used to track changes in membrane potential (Δ*ψ*_m_) in the SC, primary and secondary cells, and the *β*GA drug effects were studied. The onset of the photostimulation is indicated (stippled line). (**c**) Pearson correlation coefficients were established for the co-localization between the calcium signal and mitochondria. Correlation was established for cells that were photostimulated (control), treated with vehicle or treated with a low dose of *β*GA (50 *μ*M). No correlation was identified in cells that were photostimulated but treated with 100 or 200 *μ*M of *β*GA or were unstimulated (0% stimulation), indicating a loss of co-localization with *β*GA treatment. *χ*^2^-test was used to test for co-localization of Fluo8 and TMRM signal; non-parametric *T*-test to assess significance (*P<0.05). (**d**) Representative TMRM signals of the stimulated cell, as well as primary, secondary and tertiary cells are presented, indicating that the signal consists of a *ψ*_m_ hyperpolarization followed by a depolarization (arrows). (**e**) Latencies for the rise in mitochondrial calcium (Fluo8) as well as the hyperpolarization and depolarization phase of the mitochondrial membrane potential (TMRM) were determined to follow a consecutive pattern in all cells

First, we determined that blue-light photostimulation (488 nm) over the mitochondrial network using the built-in photo-bleaching device of the Ultraview/Vox system (Figure 1; see Material and Methods, Live-cell imaging and Blue laser stimulation for more details) leads to oxidative stress, followed by changes in mitochondrial membrane potential and intracellular calcium concentration. Continuous 1 Hz stimulation at 10% laser intensity (38 kw/cm^2^) was found to result in a rapid increase in oxidative stress determined using ROS dyes H_2_DCFDA (Figure 2a, top panel) and CRG (Figure 2a, third panel; latency: H_2_DCFDA, 0.58±0.38 s; CRG, 2.64±1.25 s, Figure 2c; Supplementary Video 1 for H_2_DCFDA and Supplementary Video 2 for CRG), followed by a change in intracellular Ca^2+^ imaged using Fluo8 (Figure 3a, top panel), localized to both the nucleus and mitochondria (latency: 3.60±0.73 s, Figure 3a, top row; Figures 3c and 4e), finally followed by a reduction in mitochondrial membrane potential imaged using TMRM (latency: 37.93±14.52 s; Figures 4c–e). These changes were not observed when the light stimulus was placed over the nucleus instead (data not shown).

### ARPE-19 cells are connected via connexin43-containing GJs

The RPE is a monolayer of connected cells, in which communication between cells is afforded by GJ made up of connexins Cx43 and Cx46.^8^ Here we confirmed that cells grown under our culture conditions form monolayers with tight junctions that uniformly label with ZO1 as well as GJs that label positive for connexin43 (Supplementary Figure S2). ARPE-19 cells have been shown by Udawatte *et al.*^8^ to exhibit intact GJ, as they mediate the spread of Lucifer yellow, and we have confirmed this observation using a puff of carbachol (data not shown), which has been shown to elicit a calcium wave via GJs in primary RPE cells.^9^

### Calcium wave is mediated via GJ communication

Calcium signaling has been investigated in RPE monolayers in response to different pharmacologic agents as well as to mechanical stimulation.^9^ Here we first examined whether the Ca^2+^ signal induced by photo-oxidative stress in the stimulated cell can be transmitted to neighboring cells, and if so, whether all six potential neighbors are equally affected.

At baseline and before stimulation, the Fluo8 signal was distributed uniformly at a low level throughout the cells (Figure 3b, 0 s). The Fluo8 signal in the stimulated cell increased rapidly within 3.60±0.73 s after starting the stimulation (Figure 3b, 20 s; Figure 3c). By 38.60±33.0 s, some of the neighboring cells surrounding the stimulated (primary) cell exhibit elevated levels of Ca^2+^ followed at 74.62±34.3 s by the secondary cells, and at 82.78 ±38.1 s by the tertiary cells (Figures 3c and 4e for a summary; Supplementary Video 1). Interestingly, the Ca^2+^ signal was not transferred uniformly to all six cells, but rather resulted in the transmission to a certain subset of cells. Typically, only 2–3 out of 6 potential primary, 2–3 out of 12 potential secondary and 3–4 out of 18 potential tertiary cells (Figure 3b; see also Figure 1 of Stalmans and Himpens^9^ for cell numbering) were found to be responsive to the central stimulus. The Ca^2+^ signals in all cells had a transient component (duration: 154.5±48.9 s) returning to an elevated plateau for the length of the recording period (Figure 3c).

As Ca^2+^ is transmitted via GJ in interconnected cells, we tested two GJ blockers, Cx43-specific 18-*β*-glycyrrhetinic acid (*β*GA) and 1-octanol, to block the cell-to-cell communications. Preincubation of the cells with *β*GA before photostimulation significantly reduced the spread of Ca^2+^ to neighboring cells at 50 *μ*M and completely prevented it at 100 *μ*M (Figure 4a). Consequently, the rise in intracellular Ca^2+^ was seen only in the stimulated cell, but did not spread to any of the neighboring cells (Figures 3b and c, and 4a and b; Supplementary Video 2). A cross-correlation analysis, determining Pearson correlation coefficient between the Ca^2+^ and TMRM signal, showed that under vehicle and insufficient *β*GA concentrations (50 *μ*M), there is significant cross-correlation, with photostimulation resulting in an increase in Ca^2+^ uptake into the mitochondria; however, this dropped to background levels, similar to the unstimulated control (Supplementary Video 3), in the presence of *β*GA ≥100 *μ*M (Figure 4c). Of importance, a 5 min washout removing *β*GA or 1-octanol from the media was allowed for complete recovery of GJ function and recovery of the calcium wave to neighboring cells (Figure 4a, bottom row; Supplementary Video 4).

### ROS signal transmission is independent of GJ communication

Redox signal transmission to neighboring cells may be indirect and mediated by the release of cytokines^10^ or exosomes,^11^ or mediated by GJs.^12^ Blue-light stimulation is known to cause oxidative stress and increase the levels of ROS, in particular hydrogen peroxide (H_2_O_2_), and has been shown to cause apoptotic cell death.^13,14^ Here we examined whether the stimulation of single cells with blue light resulted in the spread of ROS detectable with CRG and H_2_DCFDA. These two dyes are photostable, only fluorescing upon binding to their specific ROS ligand. CRG detects hydroxyl radicals and superoxide anions, whereas H_2_DCFDA detects hydrogen peroxide.

Weak CRG fluorescence was detectable in the cytosolic area before photostimulation (Figure 2a, top row). After 2.64±1.25 s from the photostimulation onset, the CRG signal shifted from the cytosolic area to the nucleus of the stimulated cell. After 19.85±8.51 s, the same change was seen in the primary neighbors (Supplementary Video 7). In comparison with CRG, H_2_DCFDA exhibited no positive signal before photostimulation (Figure 2a, third row). Upon photostimulation, the H_2_DCFDA signal increased after 0.58±0.38 s in the cytosolic area, but remained negative in the nucleus. Further, this change was seen in the secondary cells from 3.58±2.61 s onward (Figure 2c). In comparison with the transient nature of the Ca^2+^ signal (Figure 3b), the ROS signal continued to rise over the course of the recording period (600 s; Figure 2b; Supplementary Video 5).

Interestingly, when the GJ blocker *β*GA (Figure 2a, second and fourth row) was applied, while the amplitude and the kinetics of the ROS increase was reduced (Figure 2b), the spreading of the ROS signal to neighboring cells was not inhibited (Supplementary Video 6 for H_2_DCFDA and Supplementary Video 8 for CRG). Rather the ROS signal continuously rose over the course of the 600 s recording period (Figure 2b). These results suggest that, although the ROS-mediated signals can pass through GJs, it is transmitted mostly through the plasma membrane.

### Calcium levels are associated with homeostatic changes and cell death

The ultimate outcome of tissue dysfunction is cell death. Cell death appears to occur in cells that are susceptible to a cell death signal, and spares others that are more resilient. As we observed that only the Ca^2+^, but not the ROS signal transmission, was selective, we examined the hypothesis that Ca^2+^ homeostasis is involved in triggering cell death.

**Figure 5:**
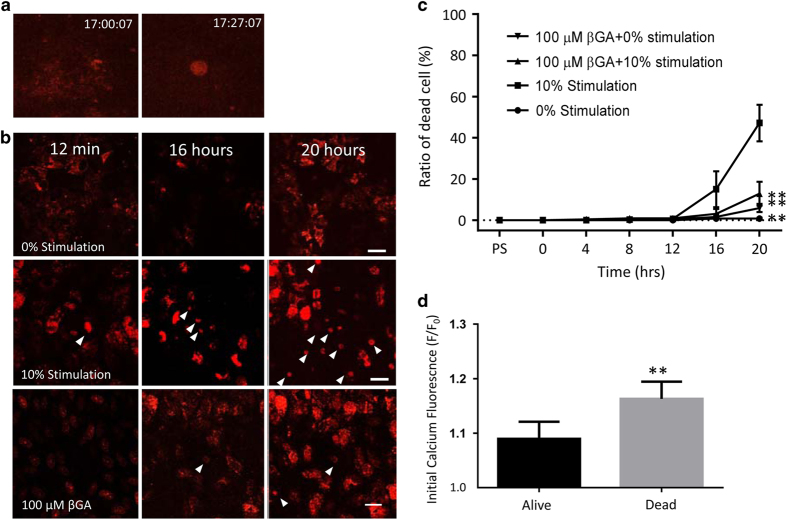
Photo-oxidative stress triggers apoptotic cell death in the cellular network requiring gap junction communication. Using the setup described in Figure 1, but using a ×40 lens, cell survival and death were monitored using a dead cell indicator TO-PRO3 over a 20-h time period. (**a**) Pyknotic cell death was triggered within 12 min in the stimulated cell. (**b**) Over the course of 20 h, there was a cytoplasmic signal based on TO-PRO-3 uptake, but there was no cell death indicated by the presence of condensed nuclei (stimulation of the central cell with the laser set to 0% power). (**c**) Over the same 20-h time course, there was significant cell death in the cellular network if the central cell was stimulated with the laser set to 10% power (same settings as Figures 2, 3 and 4). (**d**) Cell death could be prevented if gap junction communication was blocked by 18*-β*-glycyrrhetinic acid (*β*GA). Scale bar, 100 *μ*m. (**e**) The percentage of dead cells per area imaged as described in **b**–**d** is summarized for four independent experiments, confirming that *β*GA prevents cell death in a network with stimulated cells, but has no effect on cells being part of an unstimulated network. (**f**) As calcium has a role in cell death, and ROS and mitochondrial membrane potential were affected by baseline calcium levels, the baseline Fluo8 signal was compared in the population of cells that survived versus those that were dead at the 20-h time point. Cells that succumbed to death at significantly higher baseline calcium levels than those that survived (Student’s *t*-test, *P*<0.01).

Monolayers were loaded with Fluo8 and TO-PRO3 for Ca^2+^ and cell death imaging, respectively. As for the previous experiments, a single cell was stimulated at 1 Hz for 10 min (10% laser level), and imaging was continued for another 20 h in 3-min intervals. The stimulated cell exhibited the typical profile of a condensed nucleus at 12 min (Figure 5a). In stimulated cultures, cell death in neighboring cells was triggered within 16 h, resulting in condensed nuclear profiles in 47.2±9 of all cells within the imaging window by 20 h (Figures 5a and c; Supplementary Video 9). No cell death was observed in unstimulated cultures (Figures 5b and c). In addition, for all cells examined, baseline Ca^2+^ levels were obtained. On average, the cells that remained alive at the 20 h time point had a significantly lower level of Ca^2+^ at baseline, when compared with those that died (Figure 5d; see also Table 1 for statistical comparison; *P*<0.001). Interestingly, the level of Ca^2+^ at baseline was higher in cells that contained less pigmentation than those with higher numbers of granules (Figures 2d and e).

If Ca^2+^ homeostasis were to be involved in controlling cell death, other parameters such as oxidative stress and mitochondrial membrane potential would be expected to correlate with Ca^2+^ levels. A cross-correlation analysis between the baseline intensity of the Ca^2+^ signal and the induced oxidative stress/ROS CRO amplitude (Figure 2d), as well as baseline Ca^2+^ versus the induced mitochondrial membrane potential (Figure 2f) demonstrated that both the CRO (*r*=0.37, *R*^2^=0.14, *P*<0.01) as well as the *ψ*_m_ amplitude (*r*=0.28, *R*^2^=0.077, *P*<0.05) were positively correlated with baseline Ca^2+^.

### GJ communication is required for the widespread induction of cell death by phototoxicity

As GJ communication is required to mediate blue-light-induced changes in Ca^2+^, cell death in response to phototoxic stimulation was examined in the presence and absence of *β*GA. The spread of the apoptotic signal through the RPE cell population was blocked by 100 *μ*M *β*GA (Figures 5a and c), whereas *β*GA in the absence of phototoxicity had no effect on cell survival. Interestingly and importantly, the apoptotic signal did not spread radially through the RPE cell network, but rather exhibited a patchy appearance by 16 h (Figures 5a and c), which became more uniform by 20 h, suggesting that cells are differentially susceptible to the cell death signal.

### Bystander cell death triggered by local photo-oxidative stress requires ER–mitochondria Ca^2+^ transfer

Calcium homeostasis is essential for cell function and survival. To control intracellular calcium and to have it ready for release, calcium is stored within cellular organelles such as mitochondria and the ER. Calcium uptake into RPE cells is mediated by l-type calcium^15^ and TRPC channels.^16^ Ca^2+^ transfer between the cytoplasm and ER is mediated via the sarco/endoplasmic reticulum Ca^2+^ ATPase (SERCA). Its release from the ER can be mediated via the activation of either IP3 or ryanodine receptors. Uptake into mitochondria is accomplished via the mitochondrial Ca^2+^ uniporter.^17^ Here we used specific inhibitors to probe the potential mechanism of calcium-induced cell death in our model.

**Figure 6:**
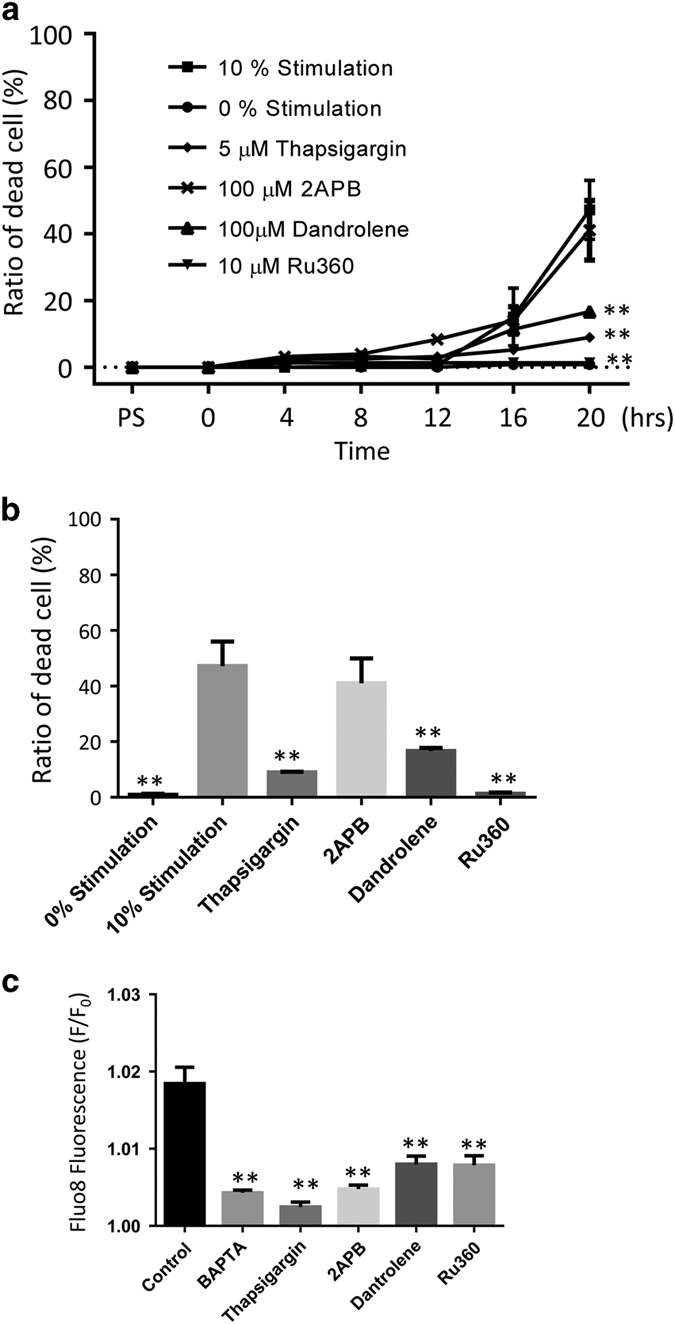
Photo-oxidative stress triggers apoptotic cell death in the cellular network requiring endoplasmic reticulum-mitochondria calcium transfer. Using the setup described in Figure 1, but using a ×40 lens, cell survival and death were monitored using a dead cell indicator, TO-PRO3, over a 20-h time period in the presence and absence of inhibitors that interfere with endoplasmic reticulum (ER)–mitochondria calcium ion (Ca^2+^) transfer. (**a**) The quantitative assessment of TO-PRO-3-positive cells after inhibitor treatment indicates that RPE networks pretreated with dandrole (ryanidine-receptor blocker), thapsigargin (SERCA pump inhibitor) or Ru360 (mitochondria calcium uptake inhibitor) were protected against photo-oxidative stress-induced cell death, whereas treatment with 2APB (IP3R blocker) had no effect. Treatment with the inhibitors alone had no effect on cell survival (data not shown). (**b**) The data presented in a were replotted for the 20-h time point. (**c**) The inhibitors all affected basal calcium levels despite their differences in effects on cell survival, indicating that the protective effect is not due to the effect on basal Ca^2+^ levels, but rather on their specific effects on ER–mitochondria Ca^2+^ transfer. Student’s *t-*test,**P<0.001.

In control cells, photo-toxic stimulation led to cell death in ~50% of the cell population within the 20 h imaging window (Figures 6a and b). This effect was reduced to 16.7±2.2% by 100 *μ*M dantrolene (ryanidine receptor blocker; *P*<0.001) and to 9.0±0.4% by 5 *μ*M thapsigargin (SERCA pump inhibitor; *P*<0.001). Likewise, cell death was completely inhibited by Ru360 (mitochondrial calcium uptake inhibitor, *P*<0.001; 1.3±0.6%). In contrast, the IP3R blocker, 2-aminoethoxydiphenyl borate (2APB), did not inhibit the cell death (41.0±1.5%; Figures 6a and b).

In addition, Ca^2+^ levels and signal spreading, as well as *ψ*_m_ were examined in these preparations. Baseline Fluo8 fluorescence was decreased by each of the drugs (Figure 6c). However, the effects of signal propagation resulting in changes in Ca^2+^ and *ψ*_m_ differed. Dantrolene did not inhibit the Ca^2+^ response or the spreading of a Ca^2+^ signal to certain surrounding cells, but the overall Ca^2+^ amplitude was lower. Also, dantrolene did inhibit *ψ*_m_ depolarization in primary cells. Thapsigargin and Ru360 treatment on the other hand inhibited both the propagation of *ψ*_m_ depolarization and the Fluo8 response, whereas the treatment with 2APB inhibited the propagation of the Ca^2+^ signal from the stimulated cell, while still resulting in *ψ*_m_ depolarization in certain surrounding cells (Supplementary Figure S3).

## Discussion

### Blue-light-triggered stress in individual RPE cells, which is transmitted to a subset of neighbors

Blue light is assumed to represent a risk factor for the development of dry AMD.^18^ In experimental systems, blue LED light has been shown to induce more damage than white or green light,^19^ and induce ROS production and lipid peroxidation in RPE cells,^20^ resulting in apoptotic cell death.^21^ Finally, intense blue light can cause mitochondrial^22^ and nuclear DNA damage.^23^

Herein we identified that repeated photo-spot stimulation at 488 nm triggered changes in oxidative stress and mitochondrial membrane potential, eliciting responses in the stimulated as well as certain connected neighbors. Importantly, stimulation of the mitochondrial network rather than the nucleus was found to be effective. The mitochondrial electron transport chain in the inner mitochondrial membrane contains photosensitive chromophores (cytochromes and porphyrins), and light absorption can alter the mitochondrial electron transport redox reaction and energy coupling. Our data are supported by the results demonstrating that blue light can alter respiration rates,^24^ lead to the generation of H_2_O_2_^25^ and trigger changes in mitochondrial ROS production that lead to a dose-dependent elevation of mitochondrial calcium.^26^

Coupling of RPE monolayers has been reported previously, triggering Ca^2+^ waves by bath application of pharmacological stimuli or mechanical perturbations.^9^ Here we demonstrated for the first time that blue-light stimulation of an individual cell can trigger the spread of information involving both GJ- and membrane-mediated communication. The changes in Ca^2+^ and mitochondrial homeostasis (*ψ*_m_) were dependent upon signal transfer via GJs, whereas the spreading of the ROS signal occurred through GJs and across the plasma membrane. This type of signaling is consistent with the concept of a cellular bystander effect.^1^

A bystander effect requiring GJ communications has been reported in response to photostimulation using a bacteriochlorophyll-based photosensitizer, which generates intracellular superoxide anions and hydroxyl radicals,^27^ and lead so GJ-mediated cell death in cells from an unirradiated area. Likewise, in ARPE-19 cells, mechanical stimulation of a single cell induced a calcium signal in neighboring cells, an effect that was inhibited by GJ blockers.^28^ However, although these and our results suggest that GJ-mediated intercellular communication is essential to trigger the calcium wave in unstimulated cells, it does not answer the question of whether the signal that passes through the GJ is calcium, or a different signaling molecule that triggers the subsequent release of calcium.

The potential for a bystander effect by the transfer of ROS across membranes has also been confirmed previously. Although H_2_O_2_ can permeate rapidly across cell membranes, its permeation is limited. Rather, H_2_O_2_ can be transported rapidly and efficiently via aquaporin channels,^29^ a channel expressed in RPE cells and involved in transepithelial water movement.^30^ It will be of great interest to determine the mechanism whereby the ROS signal is transmitted to the neighboring cells in RPE networks.

For signal transfer, our image analysis suggests a temporal sequence of oxidative stress, followed by an increase in intracellular calcium, with a slow transition into the mitochondria, followed by a transient increase in mitochondrial membrane hyperpolarization, and subsequent mitochondrial membrane depolarization. What are plausible mechanisms that mediate those responses? Hydrogen peroxide and hydroxyl radicals have been shown to increase intracellular calcium by activating the redox-sensitive cation channel, TRPM2.^31^ Hence, in our experiments, the rise in intracellular calcium in the neighboring cells might be triggered by activating ROS-triggered Ca^2+^ influx through TRP channels, rather than the GJ-mediated transport of Ca^2+^ from the stimulated cell triggering calcium-induced calcium release. Changes in *ψ*_m_ are in part mediated by calcium,^32^ with physiological concentrations of calcium increasing *ψ*_m_ and stimulating ATP production, and calcium-overload leading to Ca^2+^-induced mitochondrial permeability transition pore (MPTP) formation, triggering caspase-dependent apoptosis.^33^ Furthermore, MPTP will release cytochrome *c*, which can be transported to adjacent neighboring cells through GJs. In future studies, we will investigate whether cytochrome *c* release from depolarized mitochondria triggered by photo-oxidative stimulation is transferred into neighboring cells.^34,35^

### Intracellular calcium and cell death

Calcium levels were found not to be uniform in a resting RPE cell network. Levels were found to be negatively correlate with the amount of pigmentation present in a cell, supporting a role of melanin in the regulation of calcium homeostasis.^36^ Importantly, cells with higher calcium at resting state were more likely to die in response to the bystander effect than those with lower baseline calcium. And overall, calcium levels in unirradiated cells correlated with ROS levels.

The ER and mitochondria as organelles that store calcium, and mitochondria as a checkpoint of apoptosis have been studied extensively. Here we add to this list that cell death by the bystander effect in RPE cells requires ER–mitochondria Ca^2+^ transfer. Here we could show the involvement of the SERCA/ER ATPase, ryanodine receptors and the mitochondrial calcium uniporter (summarized in Figure 6), using specific inhibitors. Similar protective effects have been reported for thapsigargin in protecting cerebellar granule neurons against excitotoxicity,^37^ for dantrolene in reducing Ca^2+^-mediated secondary lesions in spinal cord injury,^38^ and for Ru360 in reducing infarct size in ischemia perfusion injury.^39^ 2APB, an IP3 receptor blocker, which can also prevent the release of calcium from the ER, did not inhibit cell death in our hands. 2APB has, however, been found to inhibit cell death due to a rise in calcium triggered by mechanical and hydrogen peroxide stimulation,^28,40^ suggesting that the cell death caused by photo-oxidative stress and by extrinsic hydrogen peroxide may trigger different mechanisms and/or metabolic changes in cells. Taken together, cell death induced by photo-oxidative stress requires ER–mitochondria Ca^2+^ transfer, a mechanism that includes SERCA/ER ATPase, ER efflux receptors and the mitochondrial Ca^2+^ uniporter. In the RPE cell network, the essential ER efflux receptor in this process is the RyR, although in other systems, involvement of the IP3 receptor has be identified.

**Figure 7:**
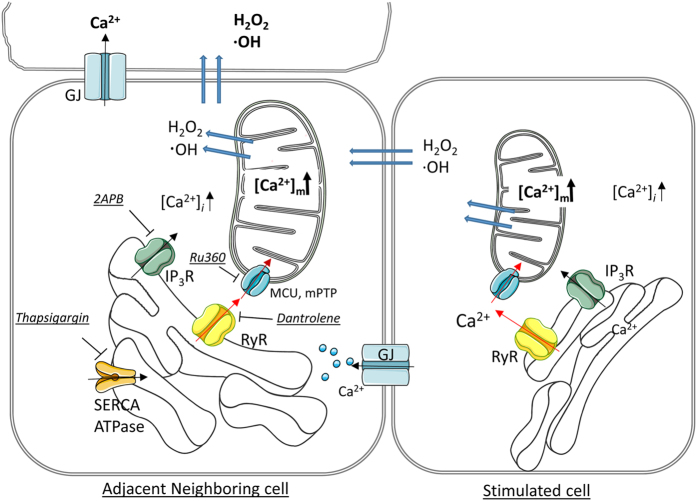
Cell death induced by photo-oxidative stress in neighboring cells is mediated by a bystander effect. In the central cell, photostimulation of the mitochondrial network leads to the generation of reactive oxygen species (ROS; H_2_O_2_, hydrogen peroxide; ·OH^−^, hydroxyl radicals), an increase in mitochondrial calcium (Ca^2+^) and a loss in mitochondrial membrane potential (Δ*ψ*_m_), leading to cell death. The transfer of the Ca^2+^ signal to neighboring cells requires gap junction (GJ) communication, whereas the transfer of the ROS signal does not. Calcium uptake in the endoplasmic reticulum (ER) is mediated by the SERCA/ER ATPase, efflux by IP3 receptors (IP_3_R) and ryanodine receptors (RyR), whereas calcium uptake in the mitochondria is mediated by uniporters or the mitochondrial permeability transition pore (MCU, mPTP), release by a Na^+^/Ca^2+^ exchanger. Here we showed that cell death by a bystander effect in RPE monolayers requires ER–mitochondria Ca^2+^ transfer utilizing RyR rather than the IP_3_R receptor

In conclusion, the results obtained by this study can be summarized as follows (Figure 7): (1) oxidative stress can be initiated in individual RPE cells using photostimulation (488 nm laser, 1 Hz), leading to rapid, consecutive changes in ROS, Ca^2+^ and *ψ*_m_ in the stimulated cell. (2) The Ca^2+^ signal could be transmitted to neighboring cells slowly and in a non-uniform way. (3) The oxidative stress signal spread fast and radially. (4) GJ blockers prevented the spreading of the Ca^2+^, but not the ROS-related signal. (5) photostimulation of a single cell triggered cell death in a subset of neighboring cells within hours. (6) Future cell death was correlated with baseline Ca^2+^ levels, and increased Ca^2+^ levels were associated with less pigmentation and a loss in *ψ*_m_. (7) Despite the continued presence of photostimulation-induced oxidative stress in the RPE network, blocking GJ communication prevented the induction of cell death. (8) Cell death could also be blocked by interfering with ER–mitochondria Ca^2+^ transfer. Together, these results demonstrate that local oxidative stress in a donor cell can trigger damage-related changes in redox and calcium homeostasis in certain connected recipient cells, a mechanism that appears to be correlated with baseline Ca^2+^ levels and pigmentation. Transfer of the ROS signal to recipient cells alone did not lead to cellular damage, rather a dual hit of ROS and the Ca^2+^-related signal was required for cellular damage. Ultimate induction of cell death appears to be dependent upon calcium transfer from the ER to the mitochondria. Finally, this metabolic signature (high baseline Ca^2+^ level) may contribute to the localized damage seen in diseases of the RPE such as age-related macular degeneration, in which initial damage seems to occur in susceptible areas, and is delayed in more resilient areas.

## Materials and methods

### Cell cultures

ARPE-19 cells (immortalized human RPE cells, passage 30–50; ATCC, Manassas, VA, USA) were expanded in DMEM including 10% fetal bovine serum (FBS) and 1% antibiotic–antifungal agents (Thermo Fisher, Waltham, MA, USA), in 25 ml or 75 ml flasks. ARPE-19 cells were chosen as, when grown as monolayers, they express all the signature genes of human RPE cells,^41^ they develop tight, adherence and GJs, and resemble an aged RPE^42^ over time. For live-cell imaging, the cells were cultured on glass-bottom dishes (Mattek, Ashland, MA, USA) and matured in medium containing 2% FBS for >2 weeks to establish well-connected monolayers and to aid in tight junction and GJ formation.^43^ For imaging, media was replaced with DMEM without phenol red (Thermo Fisher), buffered with 20 mM HEPES and supplemented with 2 mM probenecid to prevent active dye exclusion by the cells, with the pH adjusted to 7.3–7.4 using HCl.

### Immunocytochemistry

Immunocytochemistry was also performed on ARPE-19 cells grown as monolayers on transwell plates, fixed in 4% paraformaldehyde. After extensive washing, cells were incubated either in rabbit polyclonal antibodies recognizing ZO-1 (1:200; Invitrogen, Carlsbad, CA, USA), occludin (1:200; Invitrogen) or connexin43 (1:300; Sigma Aldrich, St Louis, MO, USA) in blocking solution (10% normal goat serum and 0.4% Triton-X in tris-buffered saline), and followed by Alexa Fluor 488 goat-anti-rabbit (1:500; Invitrogen) as the secondary antibody. All immunohistochemistry experiments included a no-primary antibody control (data not shown). Staining was examined via fluorescence microscopy (Zeiss, Thornwood, NY, USA) equipped with a digital black-and-white camera (Spot camera; Diagnostic Instruments, Sterling Heights, MI, USA).

### Fluorescent dyes

Imaging was performed by the following fluorescent dyes using the recommended doses according to the respective manufacturers: Fluo8 AM (2 *μ*M; AAT Bioquest, Sunnyvale, CA, USA) to indicate calcium ions; MitoTracker Deep Red (1 *μ*M; Life Technologies, Eugene, OR, USA) and MitoView 633 (1 *μ*M; Biotium, Fremont, CA, USA) to label mitochondria; and tetramethylrhodamine methyl ester (TMRM, 200 nM; Life Technologies) to indicate mitochondrial membrane potential. To detect ROS, we used CellRox Green (CRG, 2 *μ*M), CellRox Orange (CRO, 2 *μ*M) and H_2_DCFDA (1 *μ*M; all from Life Technologies). TO-PRO3 (100 nM; Life Technologies) was used as an early indicator of cell death. These dyes were applied to cells grown on glass-bottom dishes and incubated for 30 min in 37 °C in a CO_2_ incubator, followed by washing (×3 with imaging medium) before imaging.

### Drug application

GJ blocker (effective for Cx43-containing GJ), *β*GA and 1-octanol were dissolved in chloroform as a 0.1 M stock solution and applied to the cells 60 min before imaging. As Cx43 is not only found on the plasma membrane but also on the inner mitochondrial membrane, a GJ blocker that minimally affects mitochondrial membrane potential and fragmentation needed to be identified. *β*GA at concentrations higher than 150 *μ*M and 1-octanol higher than 200 *μ*M were found to be toxic to cells, whereas 100 *μ*M *β*GA and 2 mM 1-octanol were well tolerated (Figure 1). To interfere with Ca^2+^ transfer between the cytoplasm, ER and mitochondria, the following compounds were used: 5 *μ*M thapsigargin to inhibit Ca^2+^ uptake into the ER via the SERCA/ER ATPase; 100 *μ*M 2APB to block IP3 receptor-mediated Ca^2+^ release from the ER; 100 *μ*M dantrolene to block the ryanodine receptor-mediated Ca^2+^ release from the ER; and 10 *μ*M Ru360 dissolved in deoxygenated water to inhibit mitochondrial Ca^2+^ uptake via the mitochondrial Ca^2+^ uniporter.

### Live-cell imaging

For all time-lapse movies, photostimulation and image acquisition were carried out with a spinning disk confocal microscope, the UltraView VoX 3D Live Imaging System, running Volocity software (Perkin Elmer, Wokingham, UK) on a Windows 64- bit system (Figure 1). An upright microscope (Nikon Eclipse Ti) was used, collecting images with both a ×40 (apoptosis) and a ×60 lens (ROS, Ca^2+^ and TMRM imaging). For image acquisition, and to prevent artifacts based on repeated laser excitation during image acquisition, laser power was set at a maximum intensity of 0.5% for the 488, 561 and 640 nm laser lines (all at 50 mW raw laser power). Likewise, the duration of laser excitation during image acquisition was set at 200 ms to prevent sampling bias and retain good image contrast. Under these conditions, long-term imaging did not induce photo-oxidative stress (i.e., no alternations in ROS levels as determined by CRG) or changes in mitochondrial membrane potential (as determined by TMRM). Differential interference contrast images were obtained using the same system.

### Blue laser stimulation

ARPE-19 cells were stimulated with a blue spot laser (488 nm) using the built-in photo-bleaching device of the Ultraview VoX system (Figure 4). In preliminary experiments, an effective stimulation condition was established based on the following requirements: a single stimulus should not result in oxidative stress (ROS determined using CRG) or changes in mitochondrial membrane potential (using TMRM) in the stimulated cell, whereas repeated stimulation was expected to cause change. A 20 ms, 10-cycle pulse stimulation every 1 s (1 Hz) was found to produce the desired CRG and TMRM responses. Likewise, the minimum effective laser intensity was determined as 10% laser intensity (38 kw/cm^2^), resulting in reproducible effects on ROS and mitochondrial membrane potential, triggering both a response in the stimulated (primary cell) as well as triggering changes in connected (secondary, tertiary and quaternary cells). Less than 8% stimulation showed inconstant or no response in stimulated and neighboring cells. Spot size setting of ‘small circle’ (0.32 *μ*m diameter) was used and centered for stimulation on the mitochondrial network of a given cell.

### Cell death analysis

The cell death rate was calculated by triple staining the cultures with TO-PRO3, Fluo8 AM and TMRM. The TO-PRO3 dye is an apoptotic cell death indicator that is impermeant to live cells but penetrates compromised membranes and can be used to detect nuclear condensation. To analyze a larger network, cells were observed with a ×40 objective lens for 20 h and longer. The number of TO-PRO3-positive condensed nuclei were counted and calculated as a percentage of the total number of cells.

### Data analyses

To measure the fluorescent intensities of a given cell, area or organelle, a region of interest was digitally outlined using the Volocity software, and the time lapse of the corresponding intensity change was saved as a text file. The text files were read into Igor Pro 6 and the corresponding intensities, amplitudes and latencies measured (Supplementary Figure S1). Data are reported as the mean±S.E.M. Statistical significance was determined using a two-tailed Student’s *t*-test, *χ*^2^-test and ANOVA. Significance was set *P*<0.05; *n* represents the number of independent experiments.

## Figures and Tables

**Table 1:** Mitochondrial membrane potential and cell survival

*Cells*	*Stimulated (%)*	*Primary (%)*	*Secondary (%)*	*Tertiary (%)*
*Loss of *ψ*_m_*				
Dead	100.0	85.7	61.1	76.9
Alive	0.0	14.3	38.9	23.1
				
*Retention of *ψ*_m_*				
Dead	—	36.3	11.0	5.8
Alive	—	73.7	88.9	94.2

Mitochondrial membrane and cell survival or death were analyzed based on the experiments described in Figure 5. Loss and retention of mitochondrial membrane potential (as assessed by changes in TMRM signal) were significantly correlated with cell survival (alive) and cell death (dead). Cell death was based on the complete loss of TMRM fluorescence. Results from three independent experiments were averaged to obtain the percentile numbers. Each comparison (e.g., primary cell, loss of a *ψ*_m_, dead versus alive percentile) was found to be significant at *P* <0.001 using a *χ*^2^-test.

## Notes

Supplementary Video 9

Supplementary Snapshot Video 1

Supplementary Snapshot Video 2

Supplementary Snapshot Video 3

Supplementary Snapshot Video 4

Supplementary Snapshot Video 5

Supplementary Snapshot Video 6

Supplementary Snapshot Video 7

Supplementary Snapshot Video 8

Supplementary Snapshot Video 9
