# Supplementary figures and images for: Bystander effects elicited by single-cell photo-oxidative blue-light stimulation in retinal pigment epithelium cell networks
Source: Cell Death Discov. 2017 Feb 6;3:16071–. doi: 10.1038/cddiscovery.2016.71 (PMC5292780; doi:10.1038/cddiscovery.2016.71)

Supplemental Figure 1.

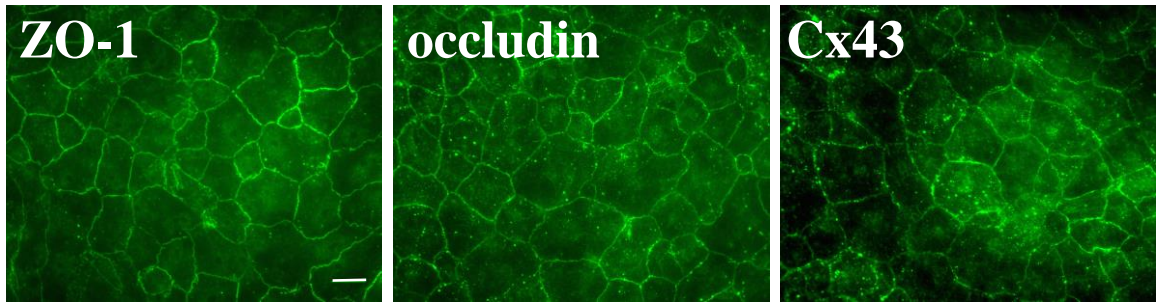

Supplemental Figure 2.

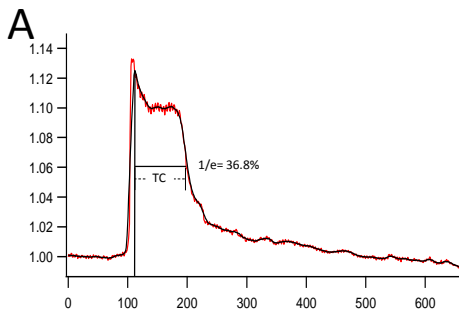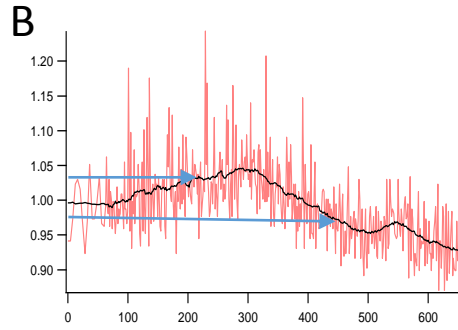

Supplemental Figure 3

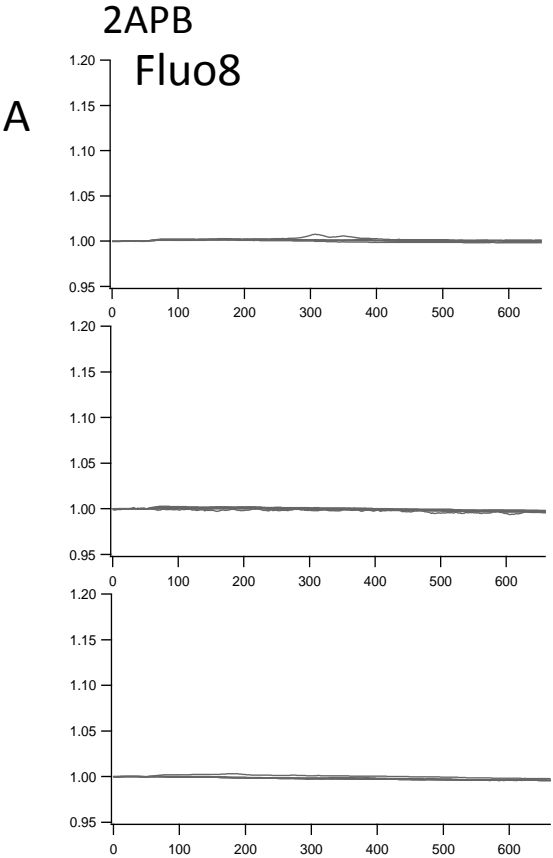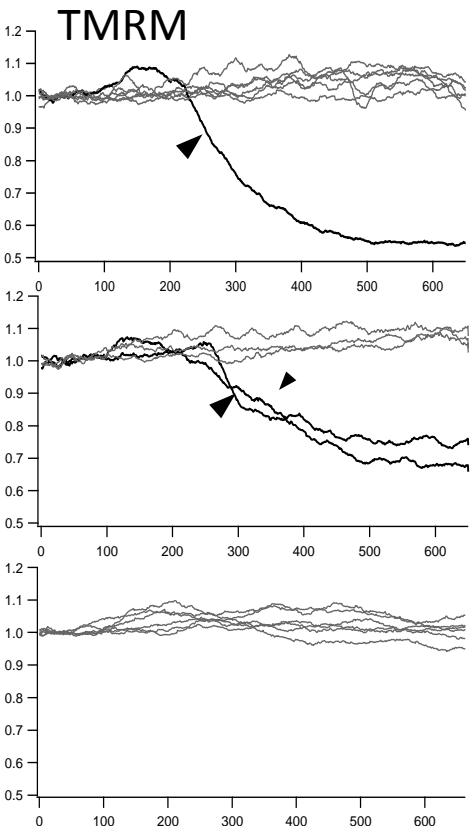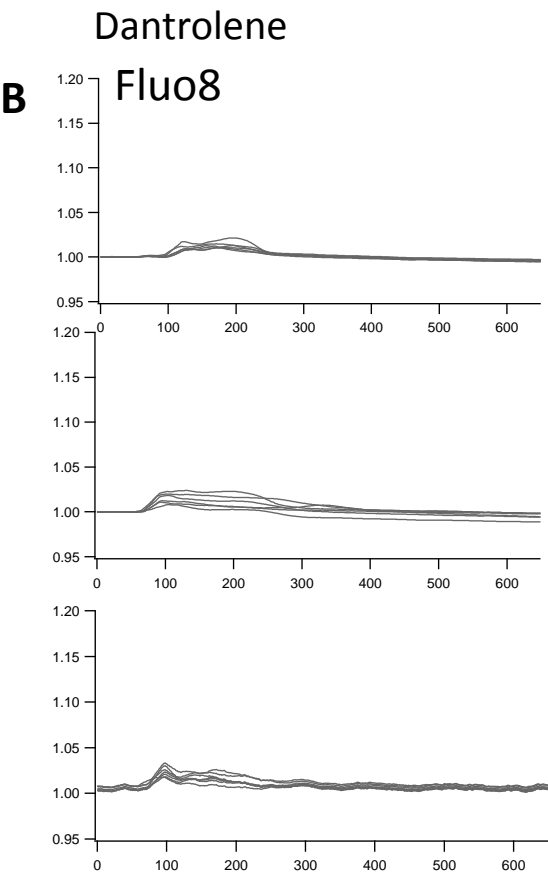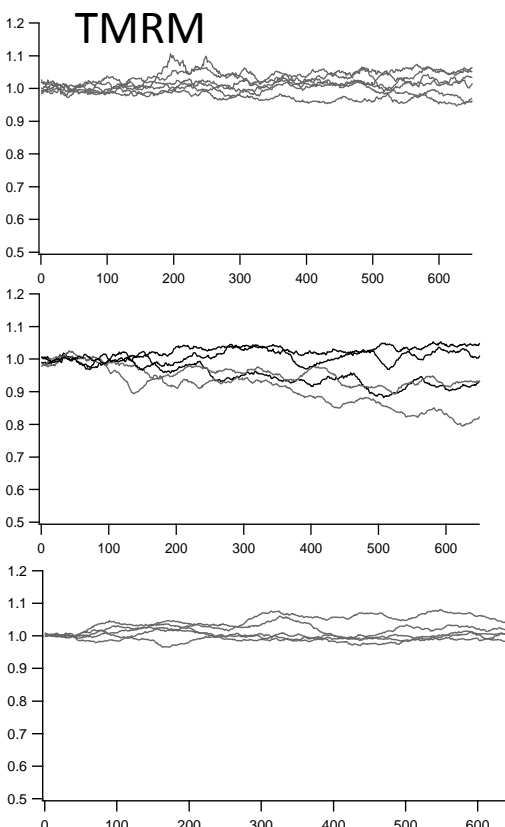

# Thapsigargin

C

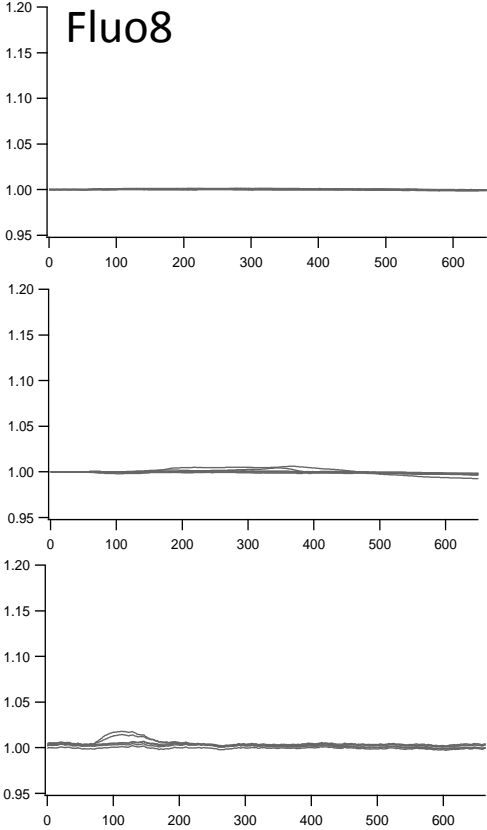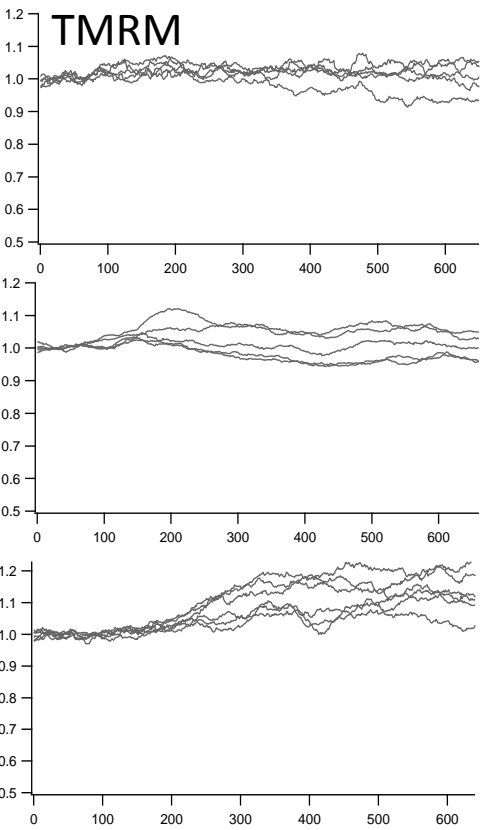

Supplement: Supplementary material [file cddiscovery201671-s1.pdf]

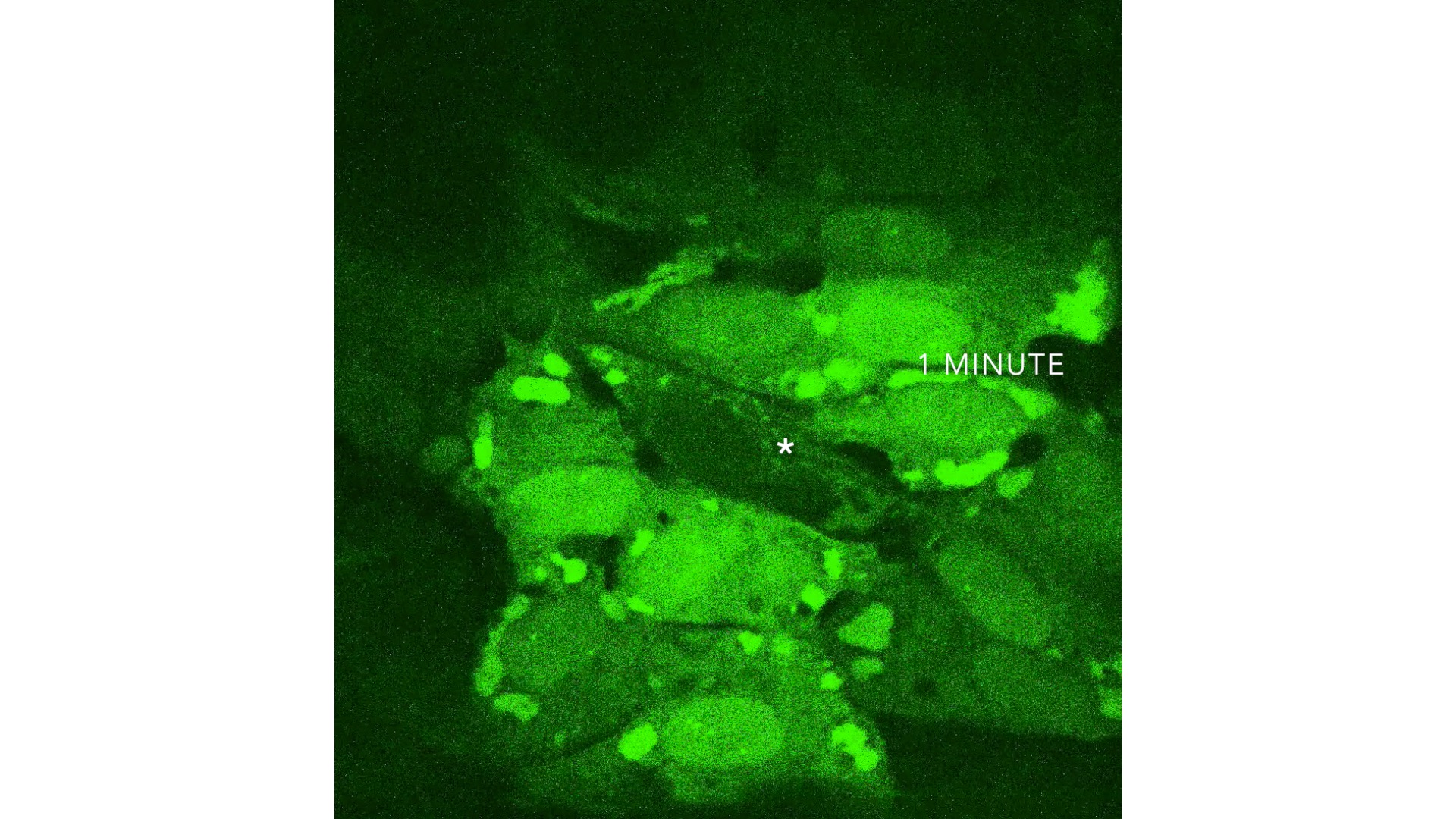

Supplement: Supplementary Snapshot Video 1 [file cddiscovery201671-s11.jpg]

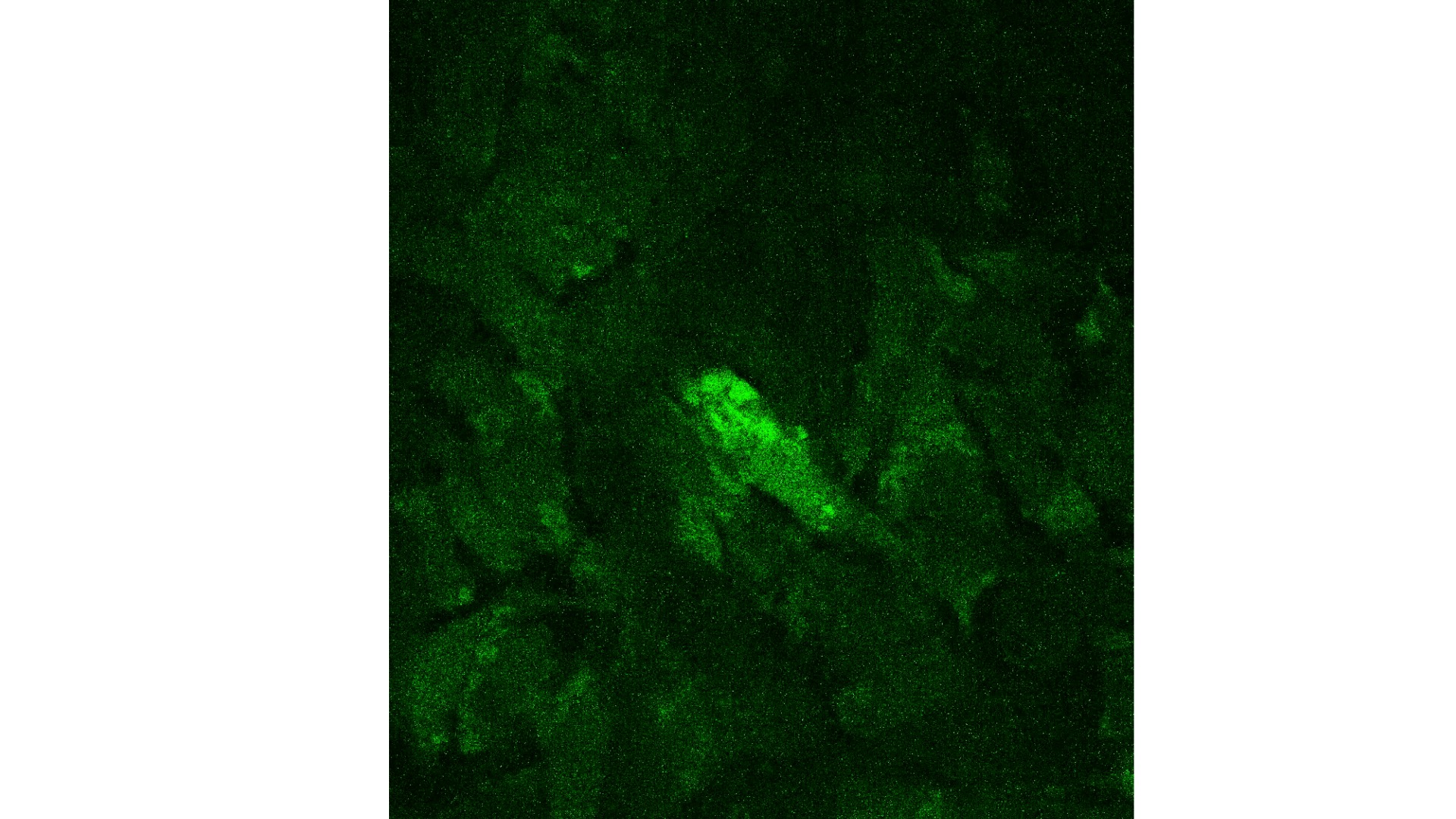

Supplement: Supplementary Snapshot Video 2 [file cddiscovery201671-s12.jpg]

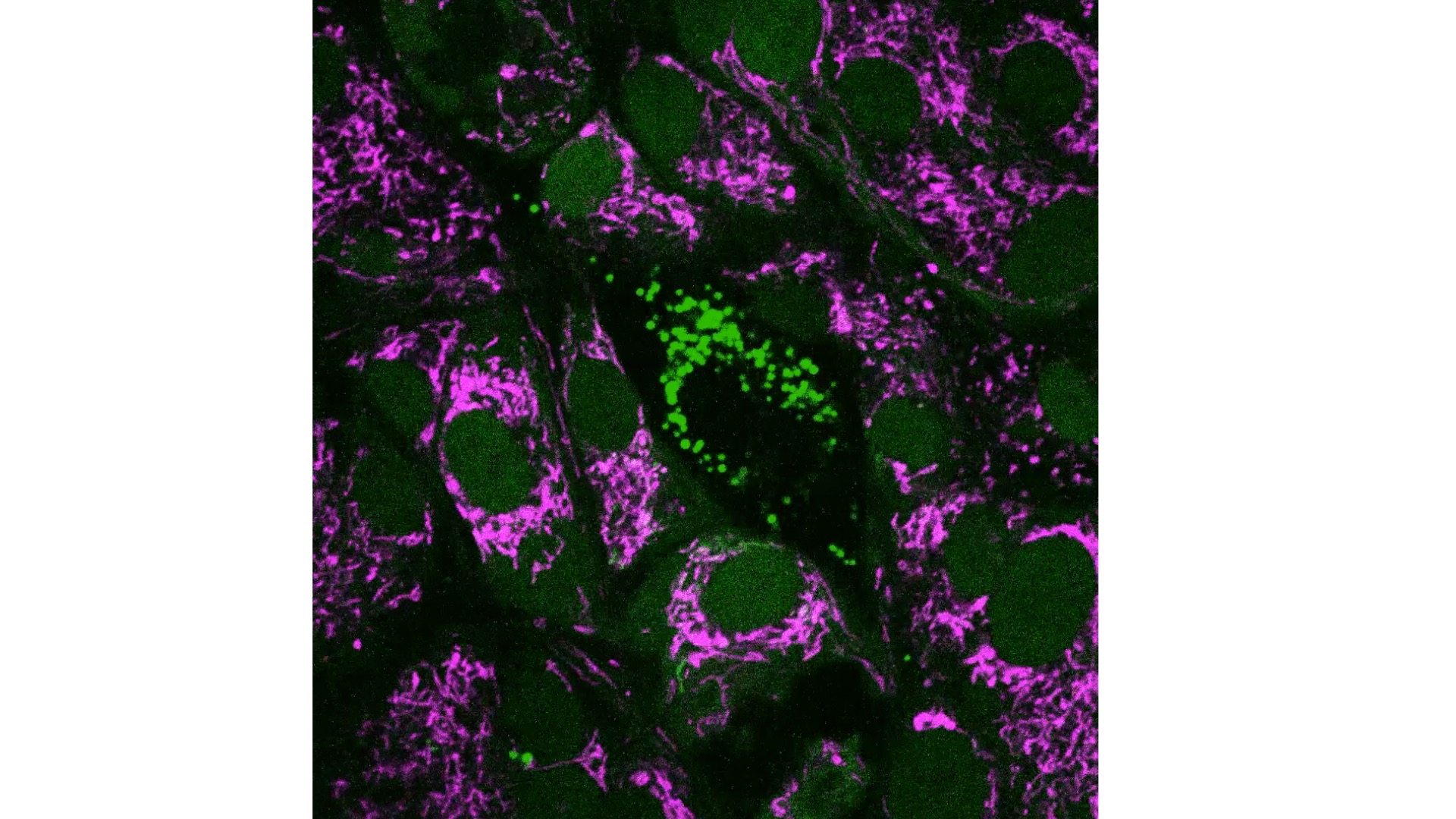

Supplement: Supplementary Snapshot Video 3 [file cddiscovery201671-s13.jpg]

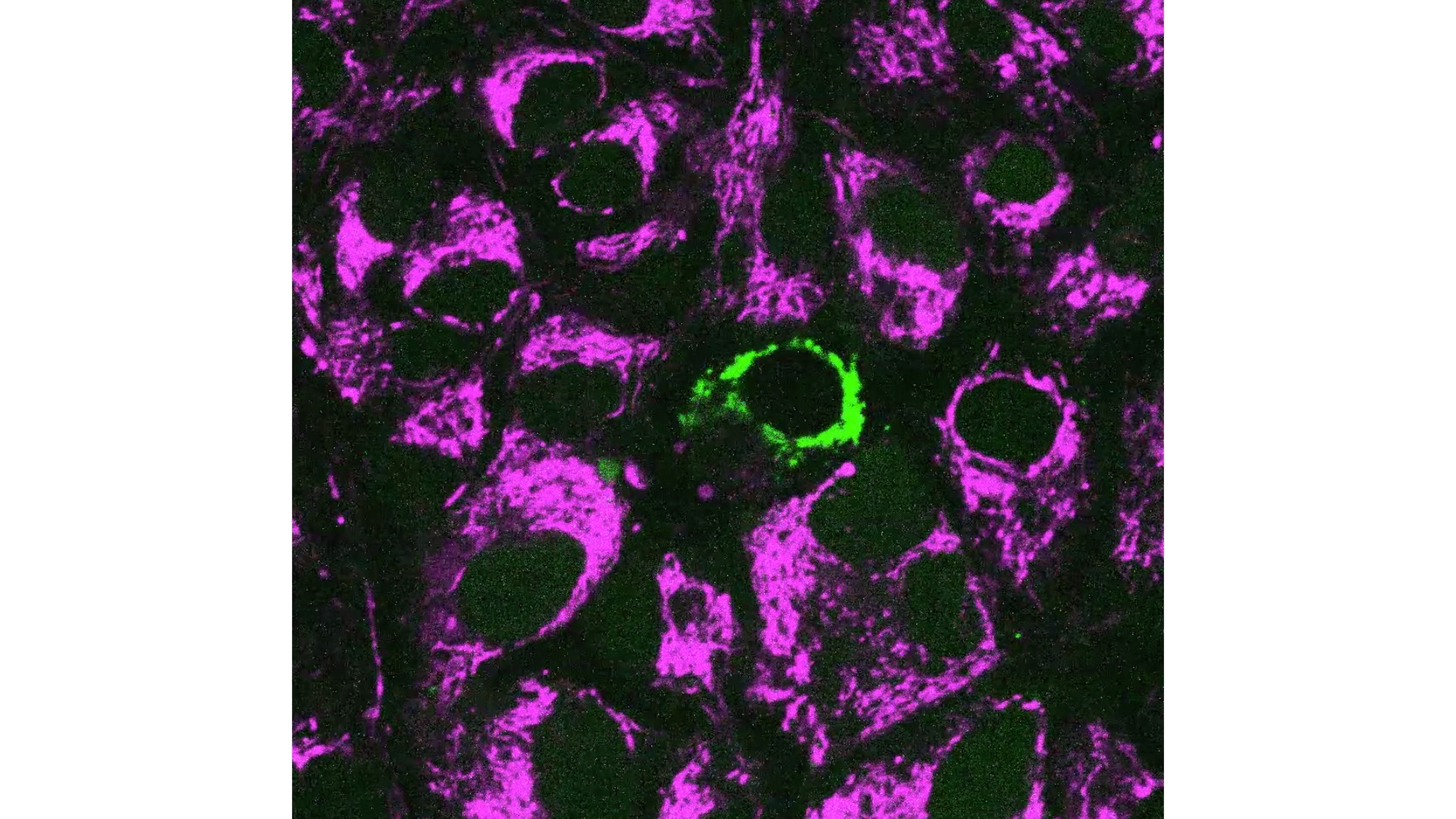

Supplement: Supplementary Snapshot Video 4 [file cddiscovery201671-s14.jpg]

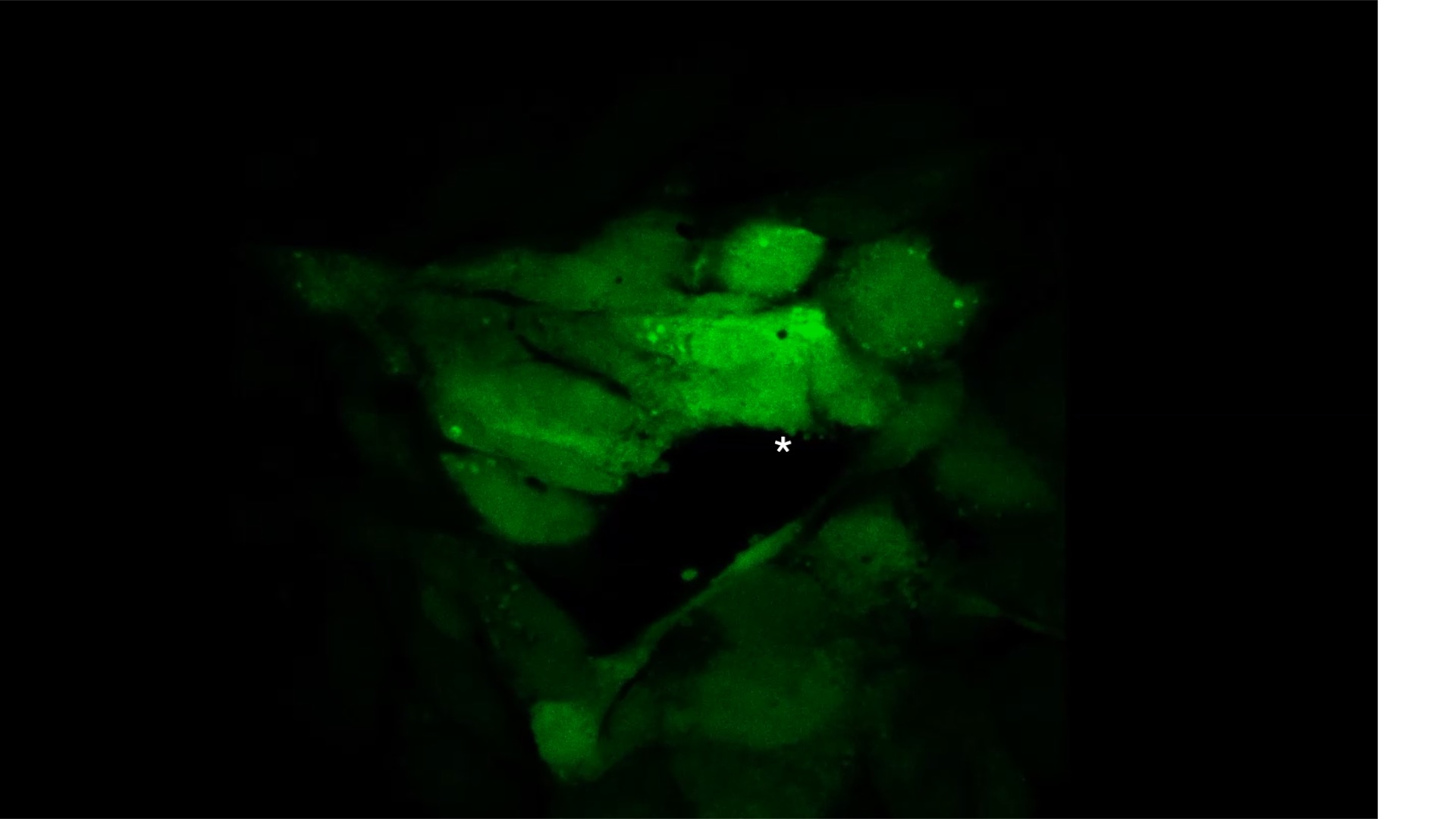

Supplement: Supplementary Snapshot Video 5 [file cddiscovery201671-s15.jpg]

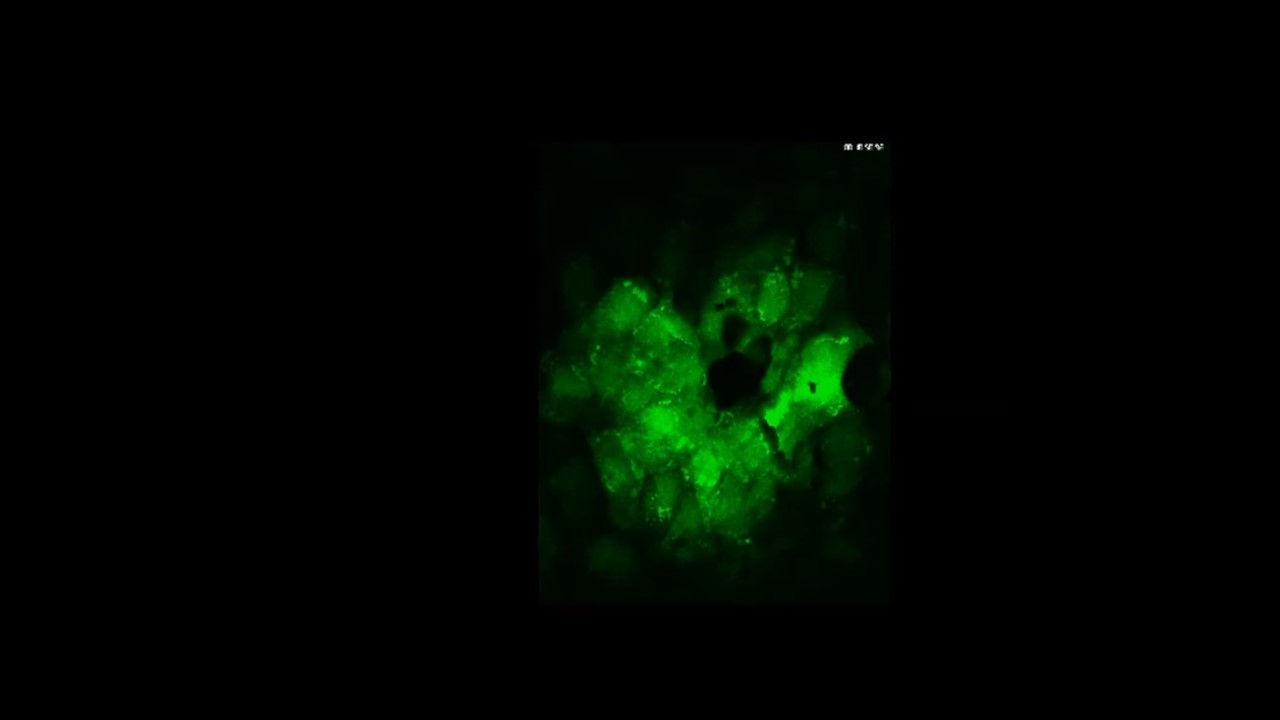

Supplement: Supplementary Snapshot Video 6 [file cddiscovery201671-s16.jpg]

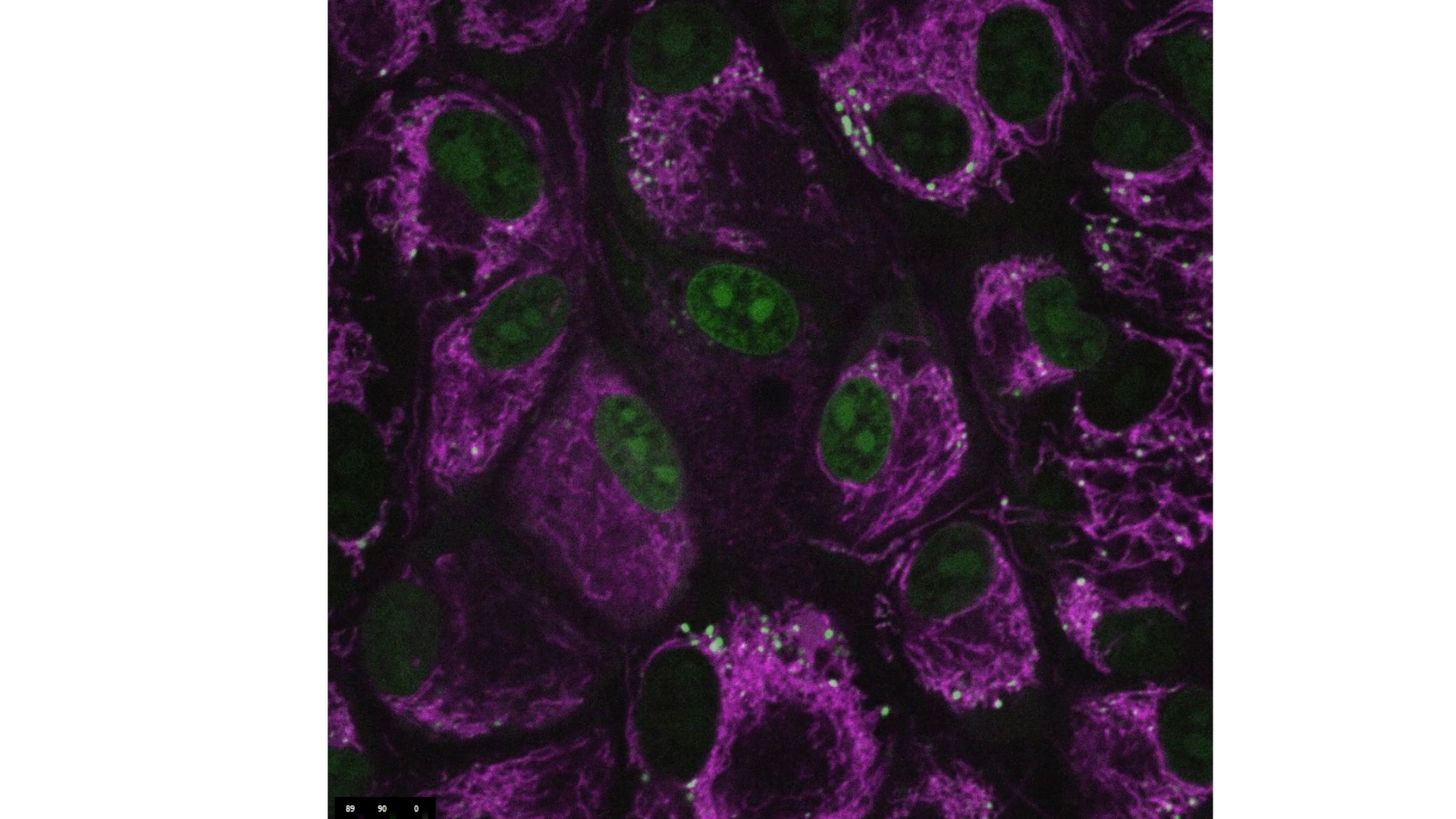

Supplement: Supplementary Snapshot Video 7 [file cddiscovery201671-s17.jpg]

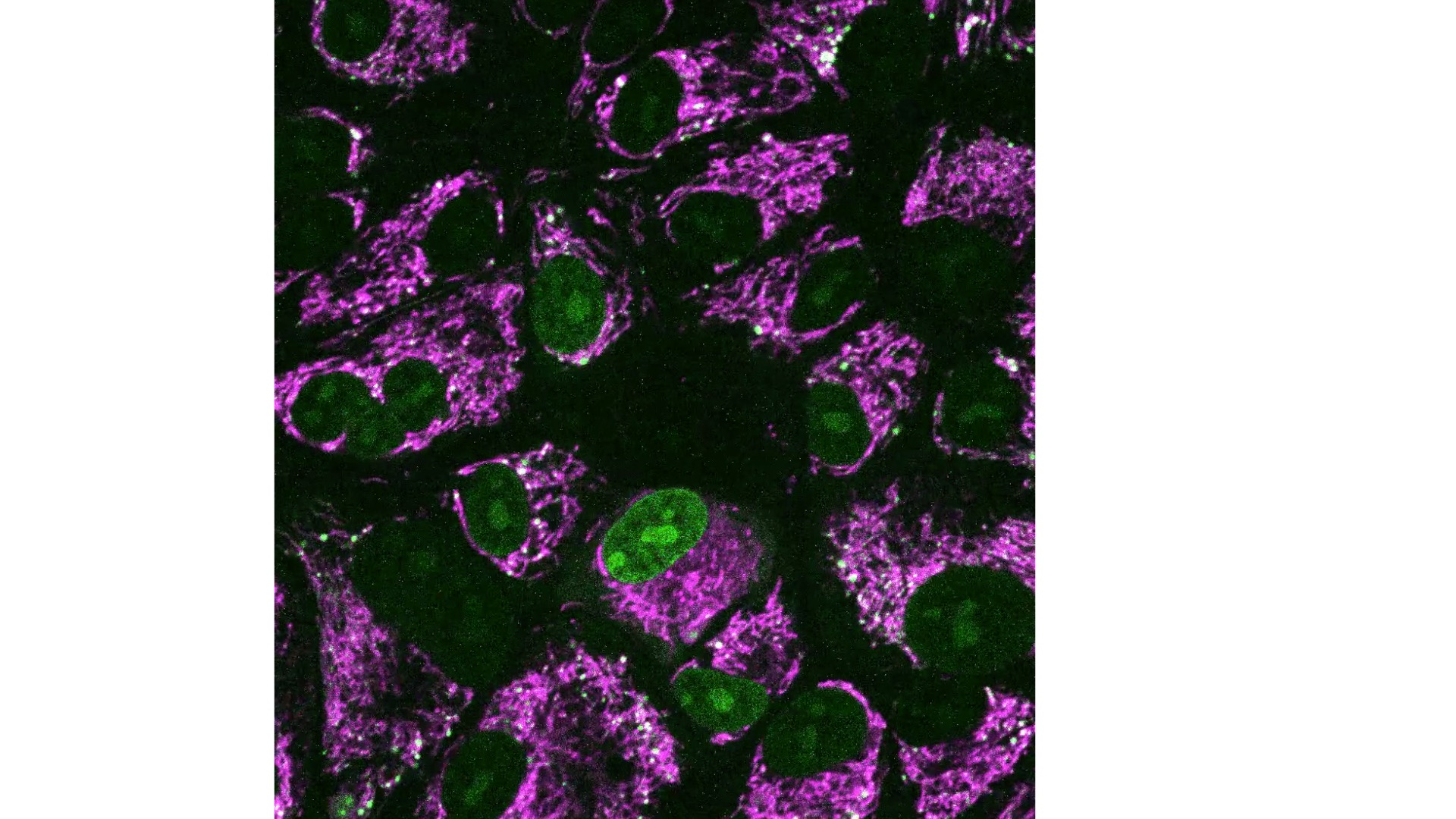

Supplement: Supplementary Snapshot Video 8 [file cddiscovery201671-s18.jpg]

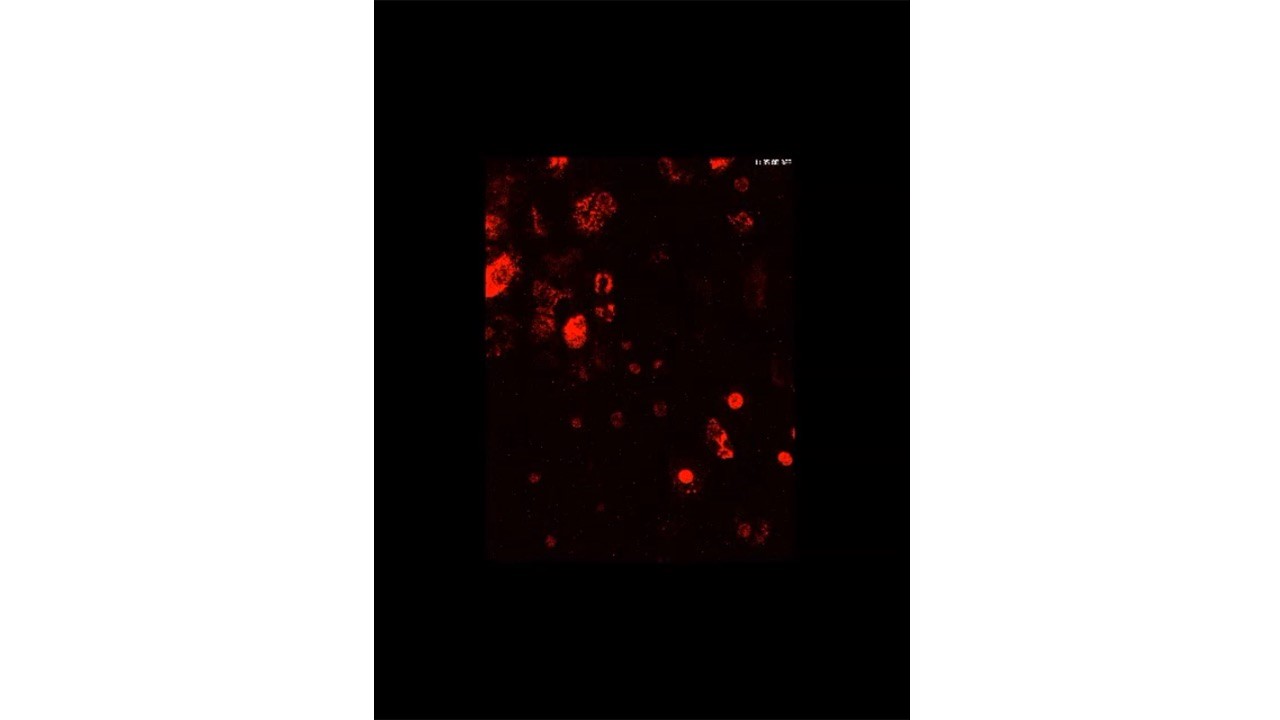

Supplement: Supplementary Snapshot Video 9 [file cddiscovery201671-s19.jpg]
